# A Phase 1C, Open Label, Single Ascending Dose Study to Evaluate the Safety, Tolerability, and Pharmacokinetics of DM199 Administered Intravenously with a Polyvinyl Chloride Bag in Adult Healthy Subjects and Adults Recently Taking Angiotensin‐Converting Enzyme Inhibitors

**DOI:** 10.1002/cpdd.1534

**Published:** 2025-04-16

**Authors:** Michael Giuffre, Annette D. Lista, Nick Paulson, Lorianne Masuoka

**Affiliations:** ^1^ Departments of Pediatrics and Cardiology Libin Cardiovascular Institute University of Calgary Calgary Alberta Canada; ^2^ DiaMedica Therapeutics, Inc. Minneapolis Minnesota USA

**Keywords:** ACE inhibitors, DM199, pharmacokinetics, phase 1C clinical trial, safety, tolerability

## Abstract

This small phase 1C, open‐label, single ascending dose study evaluated the safety, tolerability, and pharmacokinetics (PKs) of DM199, a recombinant form of human tissue kallikrein‐1 (KLK1). A small sample size of both healthy subjects and hypertensive adults recently taking angiotensin‐converting enzyme inhibitors was studied. KLK1 has a known role in vasodilation and blood flow regulation, with potential implications for treatment of acute ischemic stroke (AIS) by focally enhancing cerebral perfusion. A total of 12 subjects were enrolled; 9 healthy subjects received escalating doses of DM199 (0.1‐0.5 µg/kg), while 3 hypertensive subjects received the maximum tolerated dose of 0.5 µg/kg. Safety assessments indicated that DM199 was well tolerated, with mild adverse events reported, such as headache and flushing. No infusion‐related hypotensive events occurred, and all subjects completed the study without significant clinical issues. The study was performed following prior PK analyses revealing that DM199 exposure was greater when administered with polyvinyl chloride infusion materials compared with polyolefin infusion materials. This study supports a revised dosing strategy for DM199 in the ongoing ReMEDy2 trial for AIS and highlights the need for careful consideration of the risk‐benefit profile in the clinical context of AIS treatment.

DM199 (rinvecalinase alfa) is a recombinant form of the human tissue kallikrein‐1 (KLK1) protein, expressed from Chinese hamster ovary cells that have been transfected with a gene encoding the full‐length pre‐pro‐protein for KLK1. DM199 includes 2 specific amino acid substitutions at positions 145 and 188, which distinguish it from the native human KLK1, while preserving the disulfide bond structure of the active, mature form.

KLK1 is an endogenous serine protease involved in multiple biochemical processes, and found in blood and urine and produced predominantly by the kidney. KLK1 therapy has been studied in type 2 diabetes mellitus due to its role in the kallikrein‐kinin system, which influences insulin sensitization and glucose homeostasis by enhancing insulin‐induced glucose uptake, glucose transporter 4 translocation, and insulin receptor signaling.^1^ KLK1 also plays an important role in the regulation of vasodilation and local blood flow in the body through locally activated nitric oxide and prostaglandin availability through the release of bradykinin from low molecular weight kininogen. The most well‐characterized activity of KLK1 is the enzymatic cleavage of low molecular weight kininogen into bradykinin, which binds to bradykinin 2 receptors (B2Rs) on arterial endothelium, releasing nitric oxide, prostacyclin, and endothelial‐derived hyperpolarizing factors in the endothelial cells.^2^, ^3^, ^4^ B2Rs are upregulated in ischemic tissues, including the brain.^5^ These kinin peptides work through the cyclic guanosine monophosphate and cyclic nucleotide cyclic adenosine monophosphate pathways to preferentially relax smooth muscle cells and improve blood flow through vasodilation, potentially protecting tissues and end‐organs from ischemic damage.^6^ The ability to utilize existing compounds with a well‐documented safety profile, such as KLK1 derived from human urine (uKLK1), highlights the growing potential of systematic drug repurposing to address unmet medical needs.^7^ DM199 is currently being studied as a biological treatment for acute ischemic stroke (AIS) in patients. In China, uKLK1, has received approval from the Chinese Drug Regulatory Agency and has been commercially available as a treatment for ischemic stroke for over 15 years.^8^

This small phase 1C study was specifically undertaken in response to a Food and Drug Administration (FDA) clinical hold placed on the investigational new drug (IND) application for the DiaMedica Therapeutics, Inc. ReMEDy2 trial. ReMEDy2 is a phase 2/3 randomized, double‐blind, placebo‐controlled trial studying the use of DM199 to treat AIS. The FDA hold resulted from 3 subjects experiencing unexpected instances of clinically significant hypotension occurring shortly after beginning intravenous (IV) administration of DM199 using polyvinyl chloride (PVC) infusion materials. The acutely low blood pressure in the 3 subjects was transient, returning to their baseline blood pressure (BP) within minutes after the IV infusion was stopped with no injuries suffered or long‐lasting ill effects. Root cause investigation determined the etiology of these hypotensive events was the unintentional IV overdose of DM199. The IV overdose was determined to be caused by a switch in IV infusion materials from the previously studied polyolefin infusion materials used in early clinical trials to the PVC infusion materials used in ReMEDy2.^6^, ^7^ While there are no differences in the potency or quality of DM199 with either type of IV material, the polyolefin infusion materials showed a significant level of adsorption (binding) of DM199 in contrast to PVC material, which showed virtually no adsorption of DM199. Thus, the resulting dose given to subjects was significantly less when administered with polyolefin materials. The change in IV infusion materials to PVC in ReMEDy2 effectively resulted in an unintentional overdose of DM199, triggering the hypotensive events.

This study is aimed at assessing safety, tolerability, and pharmacokinetics (PKs) of a single dose of DM199 administered intravenously with PVC infusion materials to healthy subjects and hypertensive subjects recently taking angiotensin‐converting enzyme inhibitors (ACEIs).

## Methods

### Study Design

This was a phase 1C, open‐label, single‐center, single‐center, single ascending dose (SAD) study to evaluate safety, tolerability, and pharmacokinetics of a single dose of DM199 administered intravenously with PVC infusion materials in healthy adults (Part A) and hypertensive adults recently taking ACEIs (Part B). The study was conducted at Scientia Clinical Research Center (Randwick, NSW, Australia) on behalf of DiaMedica Therapeutics, Inc. and approved by the Bellberry Human Research Ethics Committee of the participating center. Written informed consent was obtained prior to enrollment. The study was conducted in accordance with the ethical principles that have their origin in the Declaration of Helsinki, and the protocol, and all national, state, and local laws or regulations.

### Subjects

Healthy subjects over the age of 18, weighing 50‐130 kg, with no clinically significant medical problems with no chronic medications were enrolled in Part A and hypertensive subjects over the age of 18 who had recently taken an ACEI with a last dose >24 hours prior to the start of the IV infusion were enrolled in Part B.

### Treatment

This study was conducted in 2 parts. Part A consisted of evaluating sequential cohorts of 3 healthy subjects. Each subject was given a single dose of DM199 IV diluted into 50 mL of normal saline in a PVC bag (Baxter Viaflex) and infusion materials. Each subject's participation in Part A lasted approximately 3 days from the time of dosing to the completion of all study activities. Sequential cohorts of 3 subjects received planned escalating doses of DM199 (0.1, 0.25, 0.5 µg/kg) up to 0.5 µg/kg given over approximately 50 minutes. Dose escalation assessment was based on tolerability. The safety review committee (SRC) conducted a safety review meeting when the third subject in each cohort completed their 24‐hour assessment to determine if the study could proceed to the next cohort. If any subject experienced a dose‐limiting toxicity (DLT) during their treatment, then dose escalation was stopped. If no subject experienced a DLT at a given dose level, a new 3‐subject cohort would be enrolled at an escalated dose. If one or more subjects experienced DLT at a given dose level, then an intermediate dose level was to be commenced. If one or more subjects experienced DLT at the first dose level, the study would be stopped.

The first cohort of 3 healthy subjects received 0.1 µg/kg DM199 in an infusion starting at 35 mL/h for the first 15 minutes and if tolerated, then increased to maximum of 75 mL/h to complete a 50‐mL infusion in approximately 50 minutes. If the subjects tolerated the first dose level as determined after a safety data review meeting of the SRC, the next cohort of 3 subjects received 0.25 µg/kg using the same infusion rate as for the prior dose. If these subjects tolerated this second dose level, the next cohort of 3 subjects received 0.5 µg/kg using the same infusion rate. If the 0.1 µg/kg dose level was not tolerated, the study was to be stopped. If the 0.25 µg/kg was not tolerated, then dose escalation was to be stopped, and a dose level of 0.175 µg/kg was to be tested in an additional 3 healthy subjects. If the 0.25 µg/kg was tolerated and the 0.5 µg/kg was not tolerated, then dose escalation was to be stopped, and a 0.375 µg/kg dose level was to be tested in an additional 3 healthy subjects. Alternatively, if the highest planned dose level of 0.5 µg/kg was well tolerated, then all PK data were to be reviewed and compared to the PK data from a prior DiaMedica Therapeutics, Inc. study of DM199 administered with polyolefin infusion materials in an open label, phase 1B study.

In Part B, once the maximum tolerated dose (MTD) level was determined in healthy subjects then an additional cohort of 3 hypertensive subjects received the MTD (not to exceed a total dose of 50 µg DM199) using the same infusion rate as the healthy subject cohorts. If the hypertensive subjects did not tolerate the MTD at the same infusion rates as determined after a safety data review meeting, the dose was to remain unchanged and if the initial infusion rate was 35 mL/h, the infusion rate was then to be reduced to 17.5 mL/h for the duration of the 50 mL infusion and 3 additional hypertensive subjects were to be dosed.

### Safety and Tolerability

The safety and tolerability of DM199 was evaluated in all subjects who received any amount of drug during the study. Safety analyses included a review of vital signs, including systolic and diastolic BP, heart rate (HR), respiratory rate, temperature, weight, electrocardiogram parameters, frequency distributions of abnormal physical examinations; and summaries of treatment‐emergent adverse events (TEAEs). Incidence, frequency, severity, and causality of TEAEs, incidence of serious adverse events (SAEs), and severity of infusion‐related reactions were collected and summarized. A TEAE was defined as any event not present prior to the initiation of DM199 or any event already present that worsened in either intensity or frequency following exposure to DM199. DLT included all DM199‐related with Common Terminology Criteria for Adverse Events (CTCAE) Grade 3 or higher adverse events (AEs) up to day 3. A DM199‐related TEAE was defined as AEs that were evaluated as probably or possibly related. Changes from baseline in clinical laboratory tests (hematology, clinical chemistry, coagulation, and urinalysis) were assessed using standard venipuncture techniques and monitored throughout the study.

### Pharmacokinetic and Immunogenicity Evaluation

The PK population included all subjects who received IV infusion of DM199 and had sufficient concentration data to support accurate estimation of at least 1 PK parameter. Individualized plasma concentrations, including C_max_, AUC_0‐t_, and T_max_, were summarized using descriptive statistics. Pharmacokinetic (PK) analysis of DM‐199 in human plasma was performed using a validated Meso Scale Diagnostics electrochemiluminescence immunoassay. The assay was calibrated over a range of 0.500‐32.0 ng/mL, with an anchor point at 0.333 ng/mL. Quality control samples were prepared at multiple concentration levels and met predefined validation criteria for accuracy, precision, and stability. The assay demonstrated a lower limit of quantification of 0.500 ng/mL. Stability assessments confirmed sample integrity for up to 17.25 hours at room temperature and through 3 freeze‐thaw cycles. For each assay performed, standard curves and quality control samples were run, and internal controls were required to meet predefined acceptance criteria to ensure the validity of PK and sample results. Validation was conducted in compliance with FDA bioanalytical guidelines, with results analyzed using Discovery Workbench and Watson LIMS software.

The immunogenicity population included participants who received IV infusion of DM199 and had at least 1 anti‐drug antibody (ADA) measurement. Anti‐DM199 antibodies were analyzed using a Meso Scale Diagnostics electrochemiluminescent immunoassay with a 3‐tiered approach: screening, confirmation, and titration. Calibration and quality control procedures included defined cut points (screening: 1.19, confirmatory: 33.1% inhibition, titer: 1.44). The assay demonstrated a sensitivity of 5.00 ng/mL and met all predefined acceptance criteria for accuracy, precision, and stability. Stability testing confirmed the assay's reliability for up to 21.22 hours at room temperature and through 4 freeze‐thaw cycles. The validation followed 2019 FDA guidelines and Good Laboratory Practices, with statistical analyses performed by B2S Life Sciences. For each assay performed, internal controls were required to meet acceptance criteria to ensure the validity of the data, reinforcing that assay performance was continuously monitored and controlled.

### Statistical Analysis

All statistical analyses were conducted using statistical analysis system SAS Version 9.4 (SAS Institute, Cary, NC, USA). Plasma concentration–time data were analyzed by non‐compartmental analysis using Phoenix WinNonlin Version 8.3 (Certara USA, Inc., Princeton, NJ, USA). For DM199, all values below the limit of quantification were treated as zero for the calculation of summary statistics.

## Results

### Demographics

There were 9 subjects enrolled for Part A and 3 subjects enrolled for B. The mean age was 29.9 years for Part A subjects and 52.7 years for Part B subjects. Subjects were predominantly male in Part A (77.8%) and predominantly female in Part B (100%). For both parts, the majority of subjects were White, 44.4% in Part A and 100% in Part B. All 3 subjects in Part B were taking ACEIs for hypertension. Details of the demographic characteristics of subjects and patients are available in Table S1.

### Safety and Tolerability Results

Overall, in Part A, 6 out of 9 subjects in the safety population experienced a TEAE, of which 2 subjects had DM199‐related TEAEs (Table 1). In cohort 1, DM199 0.1 µg/kg, the subjects experienced a total of 4 TEAEs that included somnolence (2 events), cognitive disorder (1 event), and musculoskeletal chest pain (1 event). Somnolence and cognitive disorder were deemed to be possibly related to DM199 and all TEAEs were Grade 1 in severity. In cohort 2, DM199 0.25 µg/kg, 2 subjects experienced TEAEs, headache and esophageal spasm. The TEAE of esophageal spasm was considered Grade 2 in severity and the TEAE of headache was considered Grade 1 in severity. None of these TEAEs were considered related to DM199 by the investigator. In cohort 3, DM199 0.5 µg/kg, 1 subject experienced catheter site pain and was considered Grade 1 in severity and was not considered to be related to DM199 by the investigator. All subjects in Part A of the study tolerated and completed dosing with MTD (0.5 µg/kg) of DM199 without any interruption in infusion. No infusion‐related reactions were observed in Part A subjects.

**Table 1:** Summary of Adverse Events in Subjects in Part A and Part B

	Part A				Part B	
Adverse events	DM199 0.1 µg/kg (n = 3)	DM199 0.25 µg/kg (n = 3)	DM199 0.5 µg/kg (n = 3)	Total (n = 9), n (%)	DM199 0.5 µg/kg (n = 3) [E]	Total (n = 3), n (%)
Any TEAE	3	2	1	6 (66.7)	3 [15]	3 (100.0)
TEAE (Grade > 2)	0	1	0	1 (11.1)	2 [4]	2 (66.7)
DM199‐related TEAE	2	0	0	2 (22.2)	3 [8]	3 (100.0)
DM199‐related TEAE (Grade > 2)	0	0	0	0 (0.0)	2 [3]	2 (66.7)
Infusion‐related reaction	0	0	0	0 (0.0)	3 [7]	3 (100.0)
Treatment‐emergent adverse events						
Nervous system disorders						
Somnolence	2	0	0	2 (22.2)	0 [0]	0 (0.0)
Cognitive disorder	1	0	0	1 (11.1)	0 [0]	0 (0.0)
Headache	0	1	0	1 (11.1)	3 [5]	3 (100.0)
Dizziness	0	0	0	0 (0.0)	2 [2]	2 (66.7)
Vascular disorders						
Flushing	0	0	0	0 (0.0)	3 [3]	3 (100.0)
Hot flash	0	0	0	0 (0.0)	1 [1]	1 (33.3)
Gastrointestinal disorders						
Esophageal spasm	0	1	0	1 (11.1)	0 [0]	0 (0.0)
Nausea	0	0	0	0 (0.0)	1 [1]	1 [33.3]
General disorders/administration site conditions						
Catheter site pain	0	0	1	1 (11.1)	0 [0]	0 (0.0)
Musculoskeletal/connective tissue disorders						
Musculoskeletal chest pain	1	0	0	1 (11.1)	0 [0]	0 (0.0)
Pain in extremity	0	0	0	0 (0.0)	1 [1]	1 (33.3)
Skin/subcutaneous tissue disorders						
Livedo reticularis	0	0	0	0 (0.0)	1 [1]	1 (33.3)
Investigations						
Blood pressure increased	0	0	0	0 (0.0)	1 [1]	1 (33.3)
Death	0	0	0	0	0	0

[E] represents the number of events.

Overall, in Part B, all 3 subjects in the safety population experienced a total of 15 TEAEs reported at the DM199 0.5 µg/kg MTD with the same infusion rate as the healthy subjects from Part A (Table 1). These 15 TEAEs were headache (5 events), dizziness (2 events), flushing (3 events), hot flush (1 event), nausea (1 event), blood pressure increased (1 event), pain in extremity (1 event), livedo reticularis (1 event). Two subjects had 4 Grade 2 TEAEs (flushing [probably related], headache [1 probably related event and 1 unrelated event], and pain in extremity [possibly related]). Two subjects had 3 DM199‐Related Grade 2 TEAEs (flushing, headache, and pain in extremity). Three subjects had 8 DM199‐related TEAEs. These 8 DM199‐related TEAEs included the 7 infusion‐related reactions. Dizziness was experienced by 1 subject and was considered by the investigator to be possibly related to DM199. Flushing was experienced by 3 subjects and all 3 events of flushing were considered as probably related to DM199 by the investigator. Headache was experienced in 2 subjects and both events were considered as probably related to DM199 by the investigator. Pain in extremity was experienced by 1 subject and the event was considered as possibly related to DM199 by the investigator. Livedo reticularis was experienced by 1 subject and the event was considered as possibly related to DM199 by the investigator. Of the 8 DM199‐related TEAEs experienced by 3 subjects, 2 subjects had 3 Grade 2 TEAEs (flushing, headache, and pain in extremity).

For both parts, the mean BP and HR were steady during and after dosing DM199 as seen in the Figures S1‐S4. One subject from Part B with a baseline BP of 160/90 mm Hg had started DM199 0.5 µg/kg MTD at 35 mL/h for 15 minutes and was well tolerated. The infusion rate was increased to 75 mL/h per protocol and approximately 9 minutes after the higher infusion rate began, the patient complained of generalized headache, right thumb ache over the thenar eminence, and was noted to have a clinically significant increase in BP to 174/91 mm Hg. The infusion was interrupted, and the patient's symptoms were resolved after 2 minutes without any intervention. After a total of 29 minutes of interruption, the infusion was restarted at 35 mL/h and after 15 minutes the rate was increased again to 75 mL/h. After 3 minutes at this higher rate, the participant experienced a generalized headache and face/neck flushing. The infusion was paused again for 1 minute and then restarted at 35 mL/h until completion. The subject's symptoms resolved rapidly, and the infusion was completed at 35 mL/h. The TEAE of BP increase was of Grade 1 intensity and considered to be unlikely related to DM199 by the investigator and a result of recent discontinuation of the subject's ACEI and the relative increase was potentially exacerbated by the patient's TEAE of limb pain. Besides this, no other abnormal vital sign measurement was considered clinically significant or reported as a TEAE by the investigator. No trends were observed in other vital sign measurements including respiratory rate, temperature and weight or abnormal physical examination or 12‐lead ECG results. In addition, none of the individual hematology, clinical chemistry, coagulation, and urinalysis values that were outside the reference ranges or abnormal were considered DM199‐related or clinically significant or reported as a TEAE by the investigator. Although not hemodynamically related, another subject from Part B experienced flushing during the IV infusion of DM199 on day 1; the TEAE of flushing began 2 minutes at the initial infusion rate of 35 mL/h and improved after 4 minutes infusion duration. After increasing the infusion rate to 75 mL/h, there was a rapid increase in the area of flushing and the subject reported feeling hot. After 4 minutes at 75 mL/h, the infusion rate was reduced to 35 mL/h without interruption. The flushing then became intermittent, and the participant was able to complete the infusion at the reduced 35 mL/h. This was also an infusion‐related reaction due to which the infusion rate was reduced. The TEAE of flushing was of Grade 2 severity. However, there was no infusion interruption during the DM199 infusion. All TEAEs resolved by the end of the study. There were no deaths, and no subjects were discontinued from the study due to a TEAE in Part A and B.

### Pharmacokinetic Results

**Figure 1:**
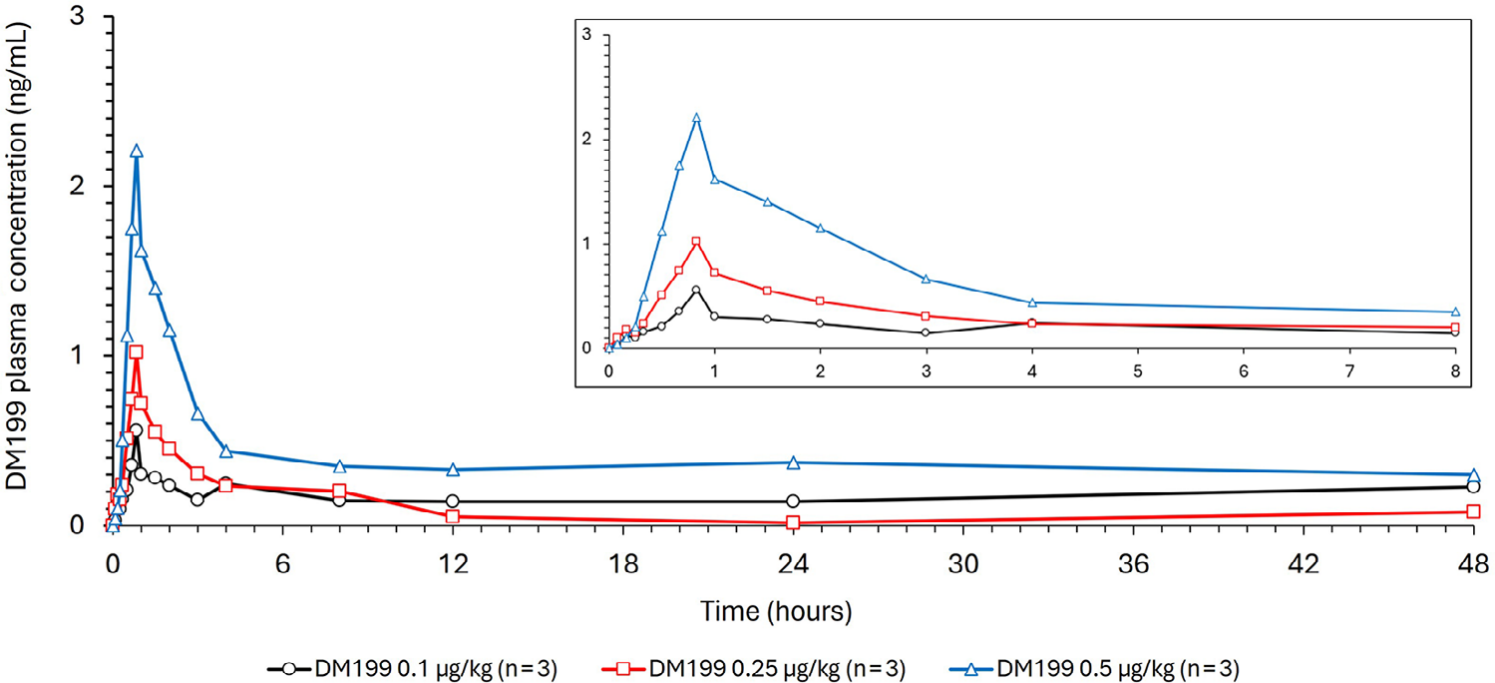
Mean plasma concentrations of DM199 baseline versus time in Part A. Mean plasma concentrations of DM199 (ng/mL) are plotted against time (hours) up to 48 hours post‐administration. Three groups in Part A are represented: DM199 0.1 µg/kg (black), DM199 0.25 µg/kg (red), and DM199 0.5 µg/kg (blue). Data points represent mean values at each time point.

**Figure 2:**
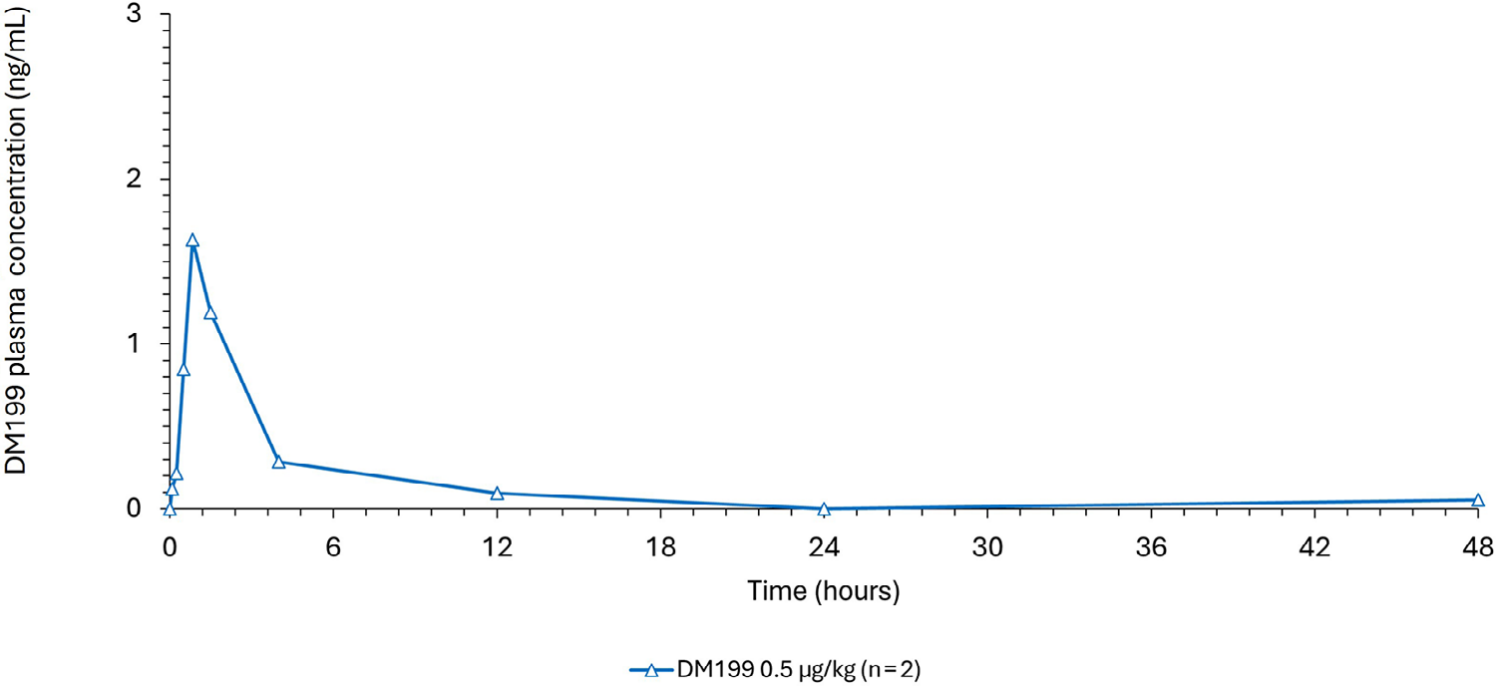
Mean plasma concentrations of DM199 baseline versus time in Part B. Mean plasma concentrations of DM199 (ng/mL) are plotted against time (hours) up to 48 hours post‐administration for the DM199 0.5 µg/kg dose group (blue). Data points represent mean values at each time point.

Plasma concentration versus time curves for DM199 are provided in Figures 1 and 2 and key PK parameters are provided in Table 2. Following single IV doses of 0.1‐0.5 µg/kg DM199 in healthy subjects in Part A, the maximum concentrations of DM199 occurred by the end of infusion in most subjects (median T_max_ = 0.83 hours). Thereafter, concentrations declined and returned to baseline predose levels at approximately 12 hours post‐dose in the majority of subjects. Following a single IV dose of 0.5 µg/kg MTD DM199 in hypertensive subjects in Part B, maximum concentrations of DM199 occurred by the end of infusion in participants (median T_max_ = 1.18 hours). Thereafter, concentrations declined and returned to predose levels at approximately 12 hours postdose. Individual DM199 pharmacokinetic parameters in Part A and Part B can be found in Table S2.

**Table 2:** DM199 Pharmacokinetic Profile in Subjects in Part A and Part B

	Part A			Part B
Parameter (units)	DM199 0.1 µg/kg (n = 3)	DM199 0.25 µg/kg (n = 3)	DM199 0.5 µg/kg (n = 3)	DM199 0.5 µg/kg (n = 2)^a^
AUC_0‐t_ (ng.h/mL), median (min, max)	1.31 (0.15, 23.6)	4.21 (3.29, 5.77)	10.8 (4.17, 42.8)	6.18 (1.15, 11.2)
C_max_ (ng/mL), median (min, max)	0.64 (0.38, 0.94)	0.91 (0.84, 1.31)	2.08 (1.85, 3.18)	1.93 (1.36, 2.49)

^a^ Pharmacokinetic evaluation in 2 subjects only.

### Immunogenicity Results

There was no significant change in plasma DM199 ADAs from baseline to postdose in all subjects. In Part A, 6 subjects (66.7%) were tested as negative for plasma antibodies to DM199 at baseline remained negative on day 3 whereas 3 subjects (33.3%) tested positive for plasma antibodies to DM199 at baseline remained positive on day 3. None of the subjects from Part A reported infusion‐related reactions. In Part B, all 3 participants (100%) tested negative for plasma antibodies to DM199 at baseline and remained negative on day 3. All 3 subjects in Part B reported at least 1 infusion‐related reaction but did not report antibodies; showing that the infusion‐related reactions were unlikely immune reactions.

## Discussion

This phase 1C study was initiated after the DiaMedica Therapeutics Inc. ReMEDy2 trial was placed on clinical hold due to 3 cases of clinically significant, transient hypotension following IV administration of DM199. Root cause analysis revealed these events were due to an unintended overdose caused by a switch from polyolefin to PVC IV infusion materials. In‐use compatibility testing demonstrated that up to half of the dose of DM199 adsorbed to the polyolefin materials which lead to an approximately 50% effective reduction in the dose administered available in Table S3. To continue ReMEDy2 and the use of IV PVC infusion materials, this phase 1C SAD study aimed to confirm a proposed revised IV dose of DM199 achieving overall exposure consistent with prior safety data, while carefully evaluating the safety and tolerability of DM199 administered IV with PVC infusion materials in both healthy subjects and hypertensive subjects recently taking ACEIs, given the potential for transient hypotensive events.

Prior to this study, an open label, phase 1B, single center, ascending dose study in healthy volunteers demonstrated safety and tolerability of IV DM199 administered via polyolefin materials. The phase 1B study was also completed to characterize the PK and pharmacodynamics after IV and subcutaneous (SC) dosing of DM199 in healthy subjects. A total of 36 subjects were enrolled. The initial component of this study was a SAD study focusing on a 30‐minute infusion of DM199 at doses of 0.25, 0.5, 0.75, and 1 µg/kg. The overall, the administration of IV DM199 up to 1.0 and 3.0 µg/kg dose via the SC route was considered safe and well‐tolerated. The second component of the study directly compared an IV infusion of 0.75 µg/kg DM199 to a 3 µg/kg SC injection. Both approaches generated similar peak plasma concentrations, but the DM199 plasma exposure was substantially longer after SC injection (up to 72 hours) compared to IV administration.^9^, ^10^

PK data from this phase 1C study were compared with PK data from the phase 1B study. The median AUC_0‐t_ for the 0.5 µg/kg dose administered with PVC infusion materials in Part A in the phase 1C study was similar to that seen with the 1.0 µg/kg dose administered with polyolefin materials in the phase 1B study (10.8 and 12.68 h•ng/mL, respectively). C_max_ for the 0.5 µg/dose in the phase 1C study and 1.0 µg/dose in the phase 1B study was also comparable (2.08 and 4.55 ng/mL, respectively). ReMEDy1, a randomized, double‐blind, placebo‐controlled, phase 2 multicenter study (NCT03290560) to assess the safety and tolerability of DM199 administered intravenously and subcutaneously in subjects with AIS was founded on these PK results. The dosing strategy of IV DM199 1 µg/kg delivered with polyolefin infusion materials followed by SC injections of DM199 3 µg/kg starting between 2 and 12 hours after the IV infusion and continuing every 72 hours (±2 hours) for the 22‐day treatment period was designed to rapidly generate presumed therapeutic concentrations of DM199 and maintain that concentration for the subsequent 22 days of treatment.

This phase 1C study demonstrates that DM199 exposure is higher when administered with PVC infusion materials compared with polyolefin materials, thereby supporting the results of the in‐use compatibility studies and supporting the proposed dosing revision of lowering the IV DM199 dose from 1.0 to 0.5 µg/kg when being administered with PVC infusion materials. Importantly, the 0.5 µg/kg IV dose of DM199 achieved similar exposure levels seen in the phase 1B study with the 1.0 µg/kg dose. For the ReMEDy2 Phase 2/3 clinical trial evaluating the treatment of AIS, the proposed IV dose of DM199 at 0.5 µg/kg, administered with PVC infusion materials, has demonstrated an acceptable safety and tolerability profile in the first 8 enrolled participants, with no evidence of hypotension observed during or after the infusion.

Given that ACEIs are widely used for hypertension and other cardiovascular conditions, understanding any potential interaction with DM199 is crucial. ACEIs inhibit the angiotensin‐converting enzyme, which can heighten bradykinin levels, while DM199 acts to further increase bradykinin production. This combined mechanism could theoretically amplify DM199's BP‐lowering effect. Testing in this subject group allowed the study to capture any synergistic effects that might impact BP control and ensure that DM199 remains safe when administered to patients whose last dose of ACEI was at least 24 hours prior to IV infusion of DM199. Based on these results, the hypertensive subjects who recently took ACEIs may not metabolize bradykinin as rapidly and may be more susceptible to the effects of a rapid increase in systemic bradykinin. Throughout the study, only 1 subject in Part B had the opposite effect, which was a clinically significant increase in BP during the DM199 infusion and BP returned to normal. This is contrary to the theoretical effect of ACEI increasing bradykinin levels and augmenting DM199's BP‐lowering effect. Importantly, no other abnormal vital signs or hypotensive events were noted. The most common infusion‐related reaction in Part B was flushing and headache, with moderate (Grade 2) severity in 2 of 3 subjects at the 75 mL/h infusion rate which improved with infusion rate reduction to 35 mL/h such that both subjects were able to complete the entire 50 mL DM199 infusion at the lower infusion rate. Therefore, hypertensive subjects recently on ACEIs may require a slower DM199 infusion rate compared to healthy subjects who were not recently taking ACEIs. All TEAEs resolved by the end of the study, no subjects were discontinued from the study due to a TEAE, and no deaths were reported. Most importantly, no subjects experienced hypotension or any drop in BP.

While DM199 demonstrated potential for further investigation to confirm its efficacy in patients with AIS, safety concerns, including the risk of transient hypotensive events, highlight the importance of careful monitoring and protocol adjustments in these patients. This study also validated the revised IV dosing rate used in the DiaMedica Therapeutics, Inc. ReMEDy2 phase 2/3 clinical trial in the treatment of AIS. Given the potential for an enhanced blood pressure‐lowering effect of DM199 in patients concurrently using ACEIs, the ReMEDy2 study protocol specifies that IV DM199 administration should be initiated at least 24 hours after the last ACEI dose. Additionally, the infusion rate for DM199 is maintained at a reduced rate of 35 mL/h throughout the infusion period to mitigate any risk of hypotensive response in these patients.

## Conclusion

In this phase 1C study, DM199 administered IV with PVC infusion materials demonstrated an acceptable safety and tolerability profile. The potential for transient hypotensive events necessitates careful dose and infusion rate adjustments, particularly in hypertensive subjects recently on ACEIs. No hypotensive events were observed, and only mild, transient adverse events were reported. Pharmacokinetic analysis revealed higher DM199 exposure with PVC infusion materials compared to previous studies with polyolefin materials, validating a revised dosing regimen of 0.5 µg/kg to achieve targeted therapeutic levels. The findings support the adjusted dosing approach used in the ongoing ReMEDy2 phase 2/3 clinical trial in AIS patients. Additionally, the study protocol adjustment of a 24‐hour interval between ACEI use and DM199 administration, along with a controlled infusion rate of 35 mL/h, mitigates the risk of any potential blood pressure‐lowering interactions in patients on ACEIs. Overall, this study establishes a safe dosing strategy for DM199. Further investigation in the ReMEDy2 trial will clarify the clinical benefits of DM199 in AIS patients. In June 2023, the clinical hold was removed on the IND application for the DiaMedica Therapeutics, Inc. ReMEDy2 clinical trial (NCT05065216).

## Conflicts of Interest

M.G.: Board of Director and shareholder of DiaMedica Therapeutics, Inc. A.L.: Employee, DiaMedica Therapeutics, Inc. N.P.: Employee, DiaMedica Therapeutics, Inc. L.M.: Employee, DiaMedica Therapeutics, Inc.

## Funding

This work was supported by DiaMedica Therapeutics, Inc.

## Supporting information



Supporting Information

## Data Availability

Data sharing is not applicable to this article.
