# Supplementary material for: A Phase 1C, Open Label, Single Ascending Dose Study to Evaluate the Safety, Tolerability, and Pharmacokinetics of DM199 Administered Intravenously with a Polyvinyl Chloride Bag in Adult Healthy Subjects and Adults Recently Taking Angiotensin‐Converting Enzyme Inhibitors
Source: Clin Pharmacol Drug Dev. 2025 Apr 16;14(6):452–60. doi: 10.1002/cpdd.1534 (PMC12130357; doi:10.1002/cpdd.1534)
Supplement: Supplementary file 1 — Supporting Information [file CPDD-14-452-s001.pdf]

Supplemental Materials

Supplemental Table 1. Baseline Characteristics

	**Part A (n=9)**	**Part B (n=3)**
**Age (years)**		
Mean (SD)	29.9 (5.0)	52.7 (6.0)
**Sex, n (%)**		
Male	7 (77.8)	0 (0.0)
Female	2 (22.2)	3 (100.0)
**Race, n (%)**		
White	4 (44.4)	3 (100.0)
Asian	3 (33.3)	0 (0.0)
American Indian or Alaska Native	1 (11.1)	0 (0.0)
Native Hawaiian or Other Pacific Islander	1 (11.1)	0 (0.0)
**Ethnicity, n (%)**		
Not Hispanic or Latino	6 (66.7)	3 (100.0)
Hispanic or Latino	3 (33.3)	0 (0.0)
**Height (cm)**		
Mean (SD)	172.2 (7.6)	165.8 (9.4)
**Weight (kg)**		
Mean (SD)	73.5 (16.3)	89.3 (15.4)
**Body mass index (kg/m²)**		
Mean (SD)	24.6 (3.6)	32.3 (3.0)
**Prior hypertension medications, n (%)**		
Perindopril 2.5 mg daily	0 (0.0)	2 (66.6)
Ramipril 5 mg daily	0 (0.0)	1 (33.3)

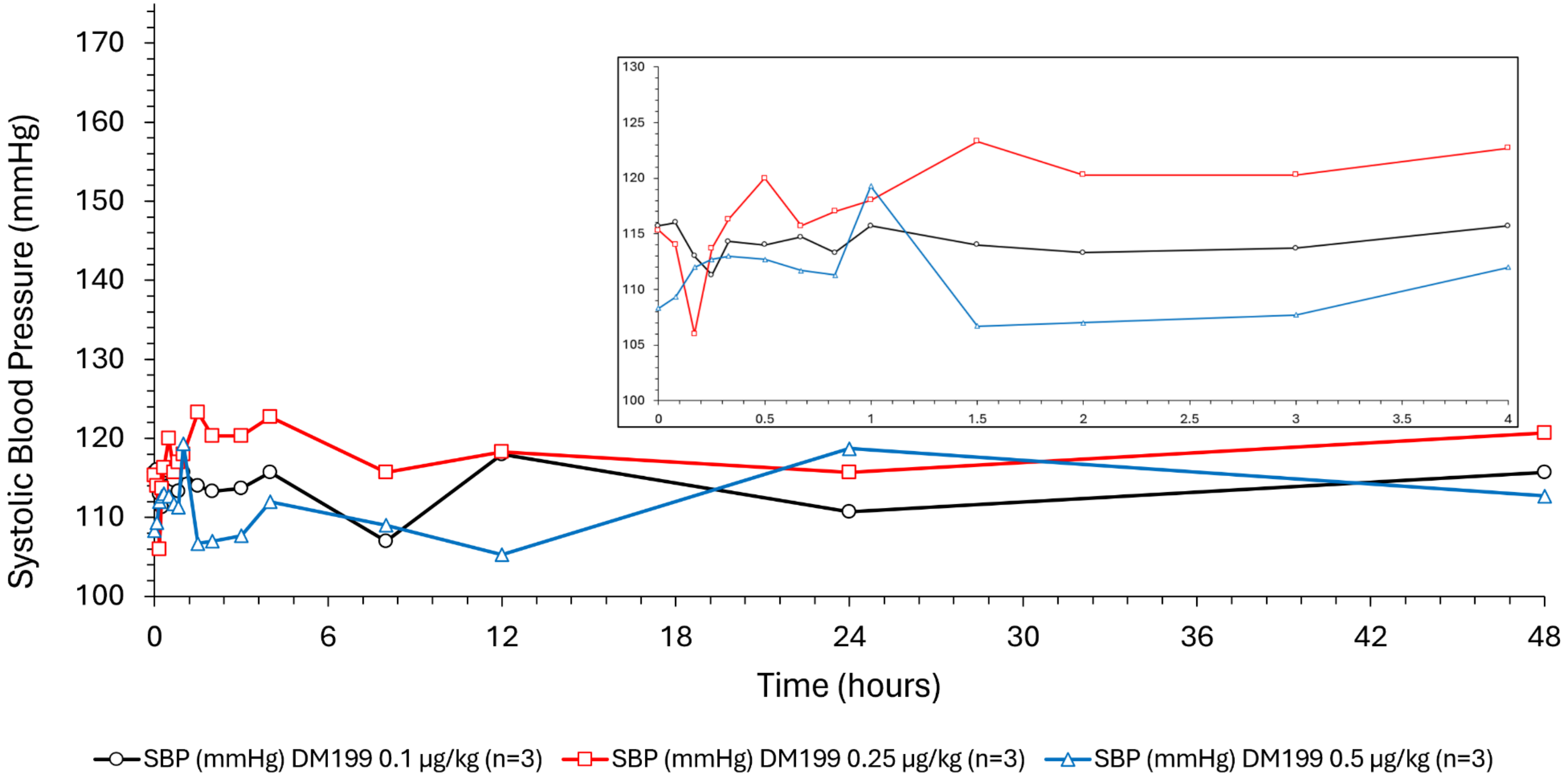

Supplemental Figure 1. Mean Systolic Blood Pressure over Time in Part A

Mean systolic blood pressure (SBP) is plotted against time (hours) up to 48 hours post-administration of DM199 in Part A. Three groups in Part A are represented: DM199 0.1 µg/kg (black), DM199 0.25 µg/kg (red), and DM199 0.5 µg/kg (blue). Data points represent mean values at each time point.

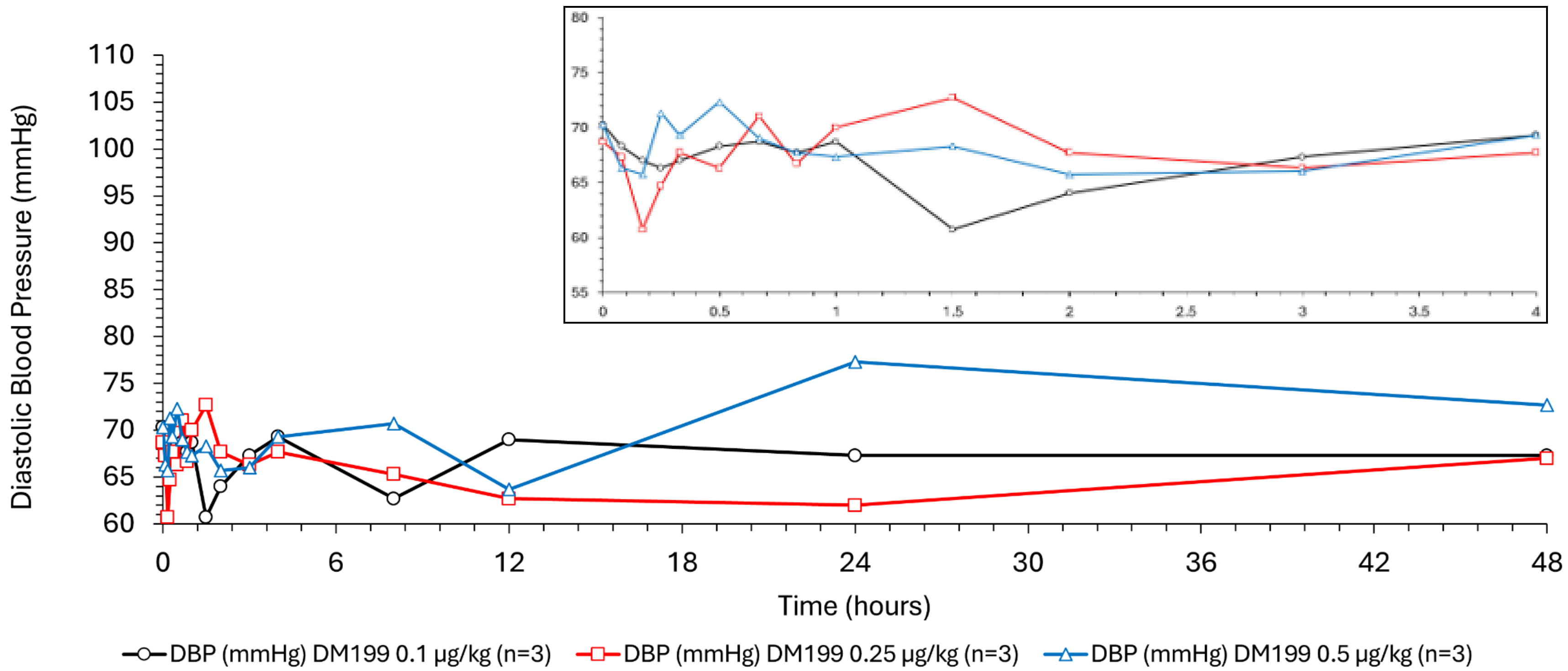

Supplemental Figure 2. Mean Diastolic Blood Pressure over Time in Part A

Mean diastolic blood pressure (DBP) is plotted against time (hours) up to 48 hours post-administration of DM199 in Part A. Three groups in Part A are represented: DM199 0.1 μg/kg (black), DM199 0.25 μg/kg (red), and DM199 0.5 μg/kg (blue). Data points represent mean values at each time point.

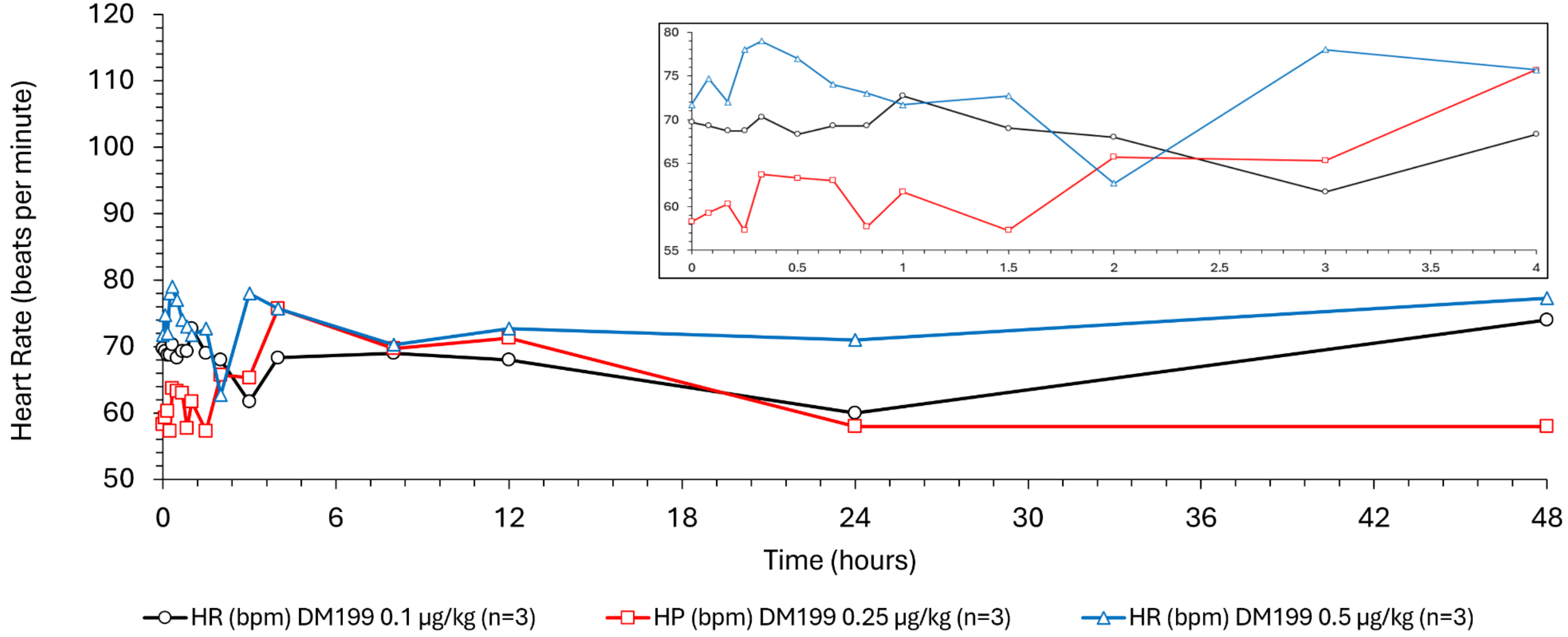

Supplemental Figure 3. Mean Heart Rate over Time in Part A

Mean heart rate (HR) is plotted against time (hours) up to 48 hours post-administration of DM199 in Part A. Three groups in Part A are represented: DM199 0.1 µg/kg (black), DM199 0.25 µg/kg (red), and DM199 0.5 µg/kg (blue). Data points represent mean values at each time point.

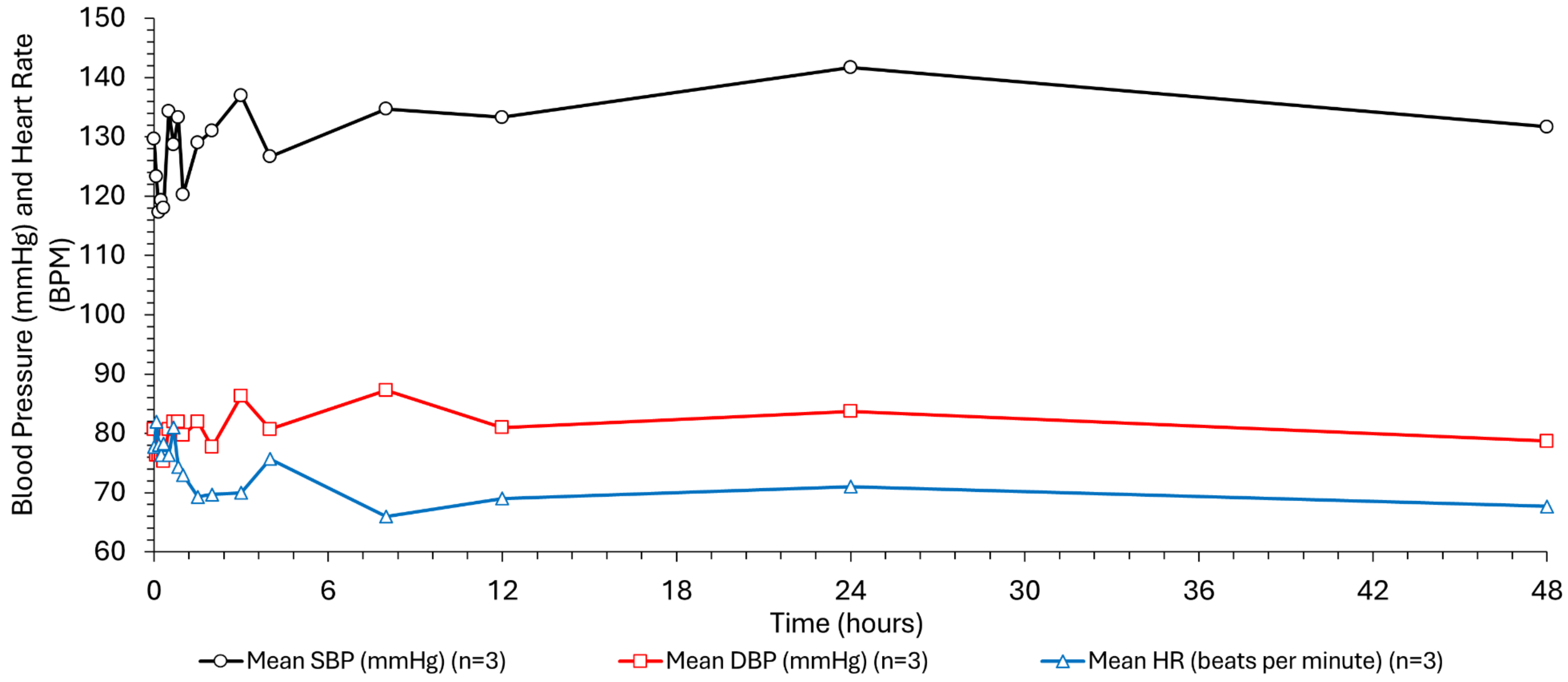

Supplemental Figure 4. Mean Systolic Blood Pressure, Diastolic Blood Pressure, and Heart Rate over Time in Part B

Mean systolic blood pressure (SBP, black), diastolic blood pressure (DBP, red), and heart rate (HR, blue) plotted against time (hours) up to 48 hours post-administration for the DM199 0.5 µg/kg dose group. Data points represent mean values at each time point.

Supplemental Table 2. Individual DM199 Pharmacokinetic Parameters in Part A and Part B

Treatment	Participant	C_{max} (ng/mL)	AUC_{0-t} (ng.h/mL)
**Part A**			
DM199 0.1 µg/kg (n=3)	101-001	0.94	23.6
	101-004	0.98	1.31
	101-005	0.64	0.15
DM199 0.25 µg/kg (n=3)	101-006	1.31	4.21
	101-007	0.84	3.29
	101-009	0.91	5.77
DM199 0.5 µg/kg (n=3)	101-016	3.18	4.17
	101-017	2.08	10.80
	101-019	1.85	42.80
**Part B**			
DM199 0.5 µg/kg (n=3)	101-021[1]	1.94	3.09
	101-023	1.36	1.15
	101-027	2.49	11.2

[1]Pharmacokinetic evaluation excluded

Supplemental Table 3. Recovery of DM199 After a One Hour Hold in Infusion Materials at Room Temperature and 40°C at 0, 8, and 32 Minutes of Infusion

Temperature	Time	Polyolefin Infusion Materials	Polyvinyl Chloride (PVC) Infusion Materials	% Recovery[1]
**Room Temperature**	**0 minutes**	0.47	0.59	78.60
	**8 minutes**	0.28	0.78	36.17
	**32 minutes**	0.47	0.75	62.30
			**Mean**	**59.02**
**40°C**	**0 minutes**	0.27	0.79	34.44
	**8 minutes**	0.43	0.89	48.81
	**32 minutes**	0.65	0.92	70.84
			**Mean**	**51.23**

[1]Percent Recovery is calculated as the percentage of DM199 recovered from polyolefin infusion materials when compared to PVC infusion materials. Polyolefin and PVC infusion materials were admixed with identical amounts of DM199 from the same stock solution, ensuring a direct comparison of recovery between the two materials.
